# Histone lysine methyltransferase SETDB2 suppresses NRF2 to restrict tumor progression and modulates chemotherapy sensitivity in lung adenocarcinoma

**DOI:** 10.1002/cam4.5451

**Published:** 2022-12-12

**Authors:** Guangda Yuan, Bowen Hu, Jun Ma, Chuanyu Zhang, Hongya Xie, Tengteng Wei, Yong Yang, Bin Ni

**Affiliations:** ^1^ Department of Thoracic Surgery The First Affiliated Hospital of Soochow University Suzhou China; ^2^ Department of Thoracic Surgery The Affiliated Suzhou Hospital of Nanjing Medical University Suzhou China

**Keywords:** chemotherapy, epigenetic regulation, LUAD, NRF2, SETDB2

## Abstract

**Objective:**

Aberrant epigenetic remodeling represents a molecular hallmark in lung adenocarcinoma (LUAD). We aim to investigate the biological roles of SETDB2 and its underlying associations with oxidative stress, providing therapeutic targets for individualized treatment of LUAD.

**Methods:**

Differential analysis was conducted via Limma package, and Kaplan–Meier analysis was performed with survival package. CCK‐8, cell proliferation assay, transwell assay, and in vivo assays were conducted to assess the function of SETDB2. Western blot assay, RT‐qPCR, and immunohistochemistry (IHC) were conducted to assess the expression levels of SETDB2/NRF2. Chromatin immunoprecipitation (ChIP) assay and ChIP‐qPCR were conducted to assess the epigenetic roles of SETDB2.

**Results:**

We found that SETDB2 expression is decreased in tumor samples versus normal tissues in TCGA‐LUAD cohort, LUAD‐EAS cohort, GSE72094 dataset, and independent Soochow‐LUAD dataset. Patients with low SETDB2 levels had a worse prognosis relative to those with high SETDB2. SETDB2 inhibition could significantly promote cell growth, migration ability, and stemness maintenance. Gene set enrichment analysis (GSEA) suggested that SETDB2 correlated with oxidative stress crosstalk and regulated NRF2 mRNA levels. ChIP assay suggested that SETDB2 mainly recruited the H3K9me3 enrichment at the NRF2 promoter region to suppress the mRNA levels of NRF2. Downregulated SETDB2 could activate NRF2 transcription and expression, thereby promoting its downstream targets, like NQO1, FTH1, and ME1. Functional experiments demonstrated that low SETDB2 allowed NRF2 to drive malignant processes of LUAD. SETDB2 overexpression attenuated the ability of NRF2 signaling to neutralize cellular reactive oxygen species (ROS) levels, leading to enhanced cell apoptosis. Overexpressed SETDB2 could inhibit tumor progression in vivo and further render LUAD cells sensitive to chemotherapy.

**Conclusions:**

In conclusion, these findings uncovered the suppressive role of SETDB2 in LUAD. SETDB2 negatively regulates NRF2 signaling to modulate tumor progression, which creates a therapeutic vulnerability in LUAD.

## 1 INTRODUCTION

Lung cancer ranks as the first cause of tumor‐related mortalities worldwide, and the 5‐year survival rate was less than 17%.^1^, ^2^, ^3^ The latest data reveal that the estimated new cases reach up to 235,760 (male 119,100, and female 116,660), and the estimated death would be 131,880 (male 69,410, and female 62,470) in 2021.^4^ Lung cancer could be classified into non‐small cell lung carcinoma (NSCLC) and small cell lung carcinoma (SCLC) based on the histological features.^5^ Besides, NSCLC could be roughly divided into lung squamous cell carcinoma (LUSC) and lung adenocarcinoma (LUAD), which is the most common subtype of lung cancer, mainly originating from bronchial epithelium.^6^, ^7^ In recent years, pivotal drivers with high frequent genetic alternations in LUAD were identified based on high‐throughput sequencing data, including KRAS mutations, EGFR activation, or ALK rearrangements. Gefitinib, one EGFR inhibitor, is regarded as the standard first‐line therapy for EGFR‐mutant advanced non‐small‐cell lung cancer (NSCLC).^8^, ^9^, ^10^ Besides, other targeted drugs (everolimus), immunotherapy (bevacizumab or nivolumab), and surgery are combined to make great progress in the treatment of LUAD.^11^, ^12^ However, the 5‐year overall survival (OS) rate still remains low with less than 20% and limited strategies are effective for terminal LUAD patients.^13^, ^14^ As a result, it is necessary to discover the in‐depth mechanisms of tumorigenesis and progression of LUAD to identify novel diagnostic and predictive biomarkers for treatment.

Epigenetic alterations mean the heritable aberrations in gene expression or cellular phenotype without altering the of DNA sequences, representing the major type of tumor‐driven events.^15^, ^16^ Notably, essential epigenetic modifiers or histone methylation factors are often found to be mutated in LUAD, including KEAP1, SMAD3, or KMT2C.^17^, ^18^ Fei Li et al. conducted the in vivo screening to identify that anti‐silencing function 1A histone chaperone (ASF1A) functions as an essential regulator of LUAD sensitivity to anti‐PD‐1 therapy, especially in Kras‐mutant tumors.^19^ Moreover, histone methyltransferase KMT2D ranks as the most highly inactivated epigenetic modifiers in LUAD, loss‐of‐function of which could promote the formation of super‐enhancers to drive glycolytic process and tumor progression.^20^ The SET domain bifurcated histone lysine methyltransferase 2 (SETDB2) mediated the formation of repressive mark trimethylated H3K9 (H3K9me3), which was reported to be associated with tumor‐related phenotypes.^21^, ^22^ Previous studies have indicated that SETDB2 could interact with and stabilize ΔNp63α to promote cancer stem cell (CSC) maintenance in breast cancer.^23^ In addition, SETDB2 inhibition promotes the sensitivity to kinase inhibitors in acute lymphoblastic leukemia (ALL), suggesting a rational target for treatment.^24^ However, few studies have been reported to elucidate the functional roles of SETDB2 in LUAD.

As is well known, transcription factor nuclear erythroid factor 2‐like 2 (NRF2) has been regarded as a master regulator that antagonizes reactive oxygen species (ROS), modulating the cellular redox balance.^25^ Previous studies have indicated that abnormal activation of NRF2 signaling is associated with various biological processes, including mitochondrial biogenesis, inflammation, and immunity.^26^, ^27^ Moreover, the associations between NRF2 and malignancies has become a research hotspot in recent years, especially in lung cancer. NRF2 regulates serine and glycine metabolism via activating transcription factor 4 (ATF4) and is linked to clinical aggressiveness in LUAD.^28^ In addition, NRF2 could also modulate the levels of exogenous nonessential amino acids (NEAAs) to promote the progression of LUAD.^29^ As previously reported, Kelch‐like ECH‐associated protein 1 (Keap1) interacts with NRF2 to restrict the protein levels of NRF2 in the cytoplasm under the normal condition.^30^ It is well known that Keap1 could function as the scaffold for Cul3‐containing E3 ubiquitin ligase, which could promote the ubiquitin–proteasome degradation of NRF2. Aberrant inactivation of Keap1, like mutations, oxidation damage, or low expression, could contribute to accumulation and subsequent activation of NRF2 in cancer.^31^, ^32^, ^33^ Apart from dysregulation of ubiquitination system, ChIP assays also found that aberrant levels of MBD2 and MeCP2 at the CpG sites of human NRF2 promoter.^34^ Therefore, whether there exist other mechanisms that contribute to NRF2 activation or suppression are meaningful questions to be answered for in‐depth understanding of the LUAD tumorigenesis.

In this study, we found that SETDB2 was a tumor suppressor in LUAD, which expressed lowly in tumor samples. We demonstrated that SETDB2 negatively regulated NRF2 transcription levels and modulated oxidative stress. This study indicated that SETDB2 possesses the potentiality to function as a novel biomarker and therapeutic target in LUAD.

## 2 METHODS

### 2.1 LUAD patients and tissue specimens

Human LUAD tumor samples (*N* = 86) matched with normal lung tissues from our hospital. All patients in this project have signed the informed consent and the study was reviewed and approved by the ethics committees of our hospital (blinded for review).

### 2.2 Cell culture and plasmid construction

The lung cancer cell lines (A549, H1299, and H522) and 293 T cells were all obtained from the American Type Culture Collection (ATCC). 293 T and H1299 cells were maintained in DMEM with 10% (v/v) FBS. H522 cells were maintained in RPMI 1640 with 10%(v/v) FBS. All cells were cultured at 37°C with 5% CO_2_.

### 2.3 Lentiviral preparation, viral infection, and stable cell generation

The pLKO.3G GFP‐shRNA plasmids were obtained from Addgene. The specific shRNA sequence of sh‐SETDB2#1: 5′‐CCGGCCCATTTCTTTCTGTAATGAACTCGAGTTCATTACAGAAAGAAATGGGTTTTTG‐3′;sh‐SETDB2#2:5′‐CCGGGCAATGATTCTAGTGAATGAACTCGAGTTCATTCACTAGAATCATTGCTTTTTG‐3′. Purinicin‐inducible GFP‐tagged lentiviral SETDB2 (Lenti‐SETDB2) were designed and synthesized by Shanghai Genechem (Shanghai, China). Cells infected with lentivirus were selected by 2 μg/mL of puromycin (Sigma, USA) to obtain stably infected cell lines. The siNRF2‐ and FAM‐labeled siNC were purchased from Guangzhou RiboBio (Guangzhou, China), and transfected.

### 2.4 CRISPR‐Cas9‐mediated gene knock out stable cell generation

For the knockout assays, pX459 plasmid was utilized to clone guide oligos to target SETDB2. The pX459 constructs were confirmed by sequencing and transfected into A549 and H1299 cells. When transfected by 24 h, the cells were treated with 1 μg/mL puromycin for 3 days. The living cells were then selected and seeded into 96‐well plate with limited dilution to obtain monoclonal cell line. We confirmed the knockout efficiency via western blot and sanger sequencing. The specific sgRNAs were listed as following: sgSETDB2#1:F: 5′‐CACCGTCCTATGCCTGTGACTCAGA‐3′, R: 5‐AAACTCTGAGTCACAGGCATAGGAC‐3′; sgSETDB2#2: F: 5‐CACCGTACAGAAATGTACAGTC TTC‐3′; R: 5‐AAACGAAGACTGTACATTTCTGTAC‐3′.

### 2.5 Cell proliferation assay, soft agar assay, and migration assay

First, cell counting kit‐8 (CCK‐8) was utilized to detect the cell proliferation rate in serial days according to the the manufacturer's protocol (Dojindo). The cells were seeded into 96‐well plate with the density of 1000 cells/well. Then, 10 μl CCK‐8 solution was put into the cell culture, and incubated for nearly 2 h, during a period of 2 to 8 days. Using a microplate absorbance reader (Bio‐Rad), the resulting color of the cells was detected and recorded at OD 450 nm. Each assay was conducted in triplicate. For the 3D soft agar colony formation assay, 10% FBS and 0.7% agar were first mixed with 2 ml gel. The cells were seeded into the medium including 10% FBS with 0.35% agar and incubated at 37°C for 3 weeks with a density of 1 × 10^5^ cells/well. The amount of soft agar colonies was analyzed and compared by ImageJ software. For the migration assay, 1 × 10^4^ cells were seeded in DMEM medium with no supplement of fetal bovine serum (FBS) into the upper chamber of each uncoated transwell. Then, the lower chamber was placed with DMEM medium with 20% FBS. After 48 h, the nonmigrating cells on the upper chamber were carefully removed by a cotton swab, while the cells underside of the filter were fixed and stained via—4% paraformaldehyde fix solution (E672002, Sangon Biotech, Shanghai, China). Matrigel invasion assays were conducted using transwell inserts (Costar) coated with Matrigel (BD Biosciences)/fibronectin (BD Biosciences). The data are presented as the mean ± SD from three independent assays.

### 2.6 Isolation of RNA and quantitative RT‐PCR

Briefly, the total RNA in tissues and cells was extracted by Trizol reagent (Invitrogen, USA), and the TOYOBO ReverTra Ace kit (TOYOBO, Japan) was utilized to conduct the reverse transcription. The mRNA expression of specific gene was quantified via quantitative reverse transcription PCR (RT‐qPCR) with Biorad CFX (Biorad, USA), in which GAPDH was selected as a control gene. The primers were designed and synthesized by Huajin Biotechnology (Shanghai, China), primer sequences were summarized as follows: SETDB2: F: 5′‐GATTTAAACCACCCCGAGA‐3′, R: 5′‐TTCATCACAAAATACCTGCT‐3′. NRF2: F: 5′‐TT GATTTAGACGGTATGCAAC, R: 5′‐TGGCATCTGAATT TAATGAGT‐3′. NQO1: F: 5′‐CTTTCAGTATCCTGCCGA GT‐3′, R: 5′‐CCAAATATTCTCCAGGCGTTT‐3′. FTH1: F: 5′‐TACGCCTCCTACGTTTACCTG‐3′, R: 5′‐AAAGAG ATATTCCGCCAAGCC‐3′. ME1: F: 5′‐CTGCTGACACGG AACCCTC‐3′, R: 5′‐CCTCTTGGCTTCCGAAACACC‐3′.

### 2.7 Chromatin immunoprecipitation (ChIP) and ChIP‐qPCR

Lung cancer cells were fixed with 1% formaldehyde and incubated at room temperature for 10 min to make DNA–protein crosslinks. Then, glycine was supplemented to stop the crosslinking process and incubated at room temperature for 5 min. A quantity of 1 ml cell lysis containing protease inhibitors (MCE, USA) was added to suspend cells and then cell lysates were sonicated to obtain 200–300 bp of chromatin fragments. Immunoprecipitation was performed with SETDB2 (1:100, Abcam, ab5517), H3K9me3 (1:50, Abcam, ab8898), and IgG (1:100, CST, 2729 S). The chromatin DNA was extracted using a DNA purification kit (TIANGEN, China) and the specific primers of NRF2 promoter were used for PCR. The primer sequences were listed as the following: NRF2: F 5′‐CCCTGCTGAGTAATCCTTTCCCGA‐3, R: 5′‐ATGTCCCGACTCCAGACTCCA‐3′.

### 2.8 Immunohistochemical staining

Lung adenocarcinoma tumors and matched benign tissues were processed into microarrays. First, tissue sections were incubated with 3% H_2_O_2_ for 15 min at room temperature. Then, slides were blocked with goat serum for 1 h and incubated with primary antibodies against SETDB2 (1:100, Abcam, ab5517) and NRF2 (1:200, Cell Signaling Technology, CST#D1Z9C). Afterward, the sections were then washed three times in 1× PBS and incubated with goat‐anti‐rabbit IgG secondary antibodies (Fuzhou Maixin Biotech). After being washed three times in 1× PBS for 5 min each, the sections were further incubated with streptavidin‐conjugated HRP (Fuzhou Maixin Biotech). Images were acquired using an Olympus camera and matched software. IHC staining was scored by two independent pathologists.

### 2.9 Western blot assay

The cells were mainly lysed in RIPA buffer (Beyotime) added with protease inhibitors cocktail (MCE, New Jersey, USA). Then, the cell lysates or immunoprecipitates were subjected to SDS–PAGE. Proteins were transferred onto the nitrocellulose membranes (GE Healthcare). Afterward, the membranes were mainly blocked with 5% nonfat milk and then incubated with the specific primary antibody overnight at 4°C. After being washed in TBS containing 0.1% Tween 20 for three times in 15 min, the membranes were incubated with the secondary antibody for 1 h under the room temperature. The ECL chemiluminescence system was utilized to visualize the binding location. The β‐actin was used as the internal control. The antibodies for western blotting assay were listed as the following: SETDB2 (Abcam, ab5517); NRF2 (Cell Signaling Technology, CST#D1Z9C); and β‐actin (Abcam, ab8226).

### 2.10 Apoptosis assay and caspase 3/7 activity assay

After indicated treatments, both suspension and attached cells were collected gently. Cell density was adjusted to 5 × 10^6^ cells/ml. A quantity of 100 ml cell suspension were incubated with 5 ml AnnexinV/FITC for 10 min and then 5 ml propidium iodide (PI) (BD Pharmingen) for 5 min at room temperature in the dark. The rate of apoptosis was measured by flow cytometry (FCM). For the detection of caspase 3/7 activity, after being treated in each group, cells were subjected to the caspase 3/7 activity assay by Caspase‐Glo 3/7 Assay Systems (Promega) according to its manufacturer's instructions. The caspase 3/7 activity was measured in triplicates and repeated independently for three times, which was represented as a fold‐increase of fluorescence calculated by comparing cells with untreated control cells.

### 2.11 Animal experiments

All experimental experiments were reviewed and approved in advance by the Ethics Review Committee for Animal Experimentation and blinded for peer review. The BALB/c nude mice were purchased from SLAC Laboratory Animal Co., Ltd. The mice were bred and maintained in mouse facilities. Afterward, 1 × 10^7^ indicated lung cancer cells were suspended in 100 μl of 1 × PBS and injected into the flanks of male nude mice. At the end of 5 weeks, mice were all sacrificed. The serial tumor volumes of solid tumors were recorded and analyzed.

### 2.12 Bioinformatic methods

The differential analysis was conducted via Limma package. The Kaplan–Meier analysis was conducted via *Survival* package. GSEA software was obtained from the GSEA home (http://software.broadinstitute.org/gsea/index.jsp), which was supported by JAVA 8 platforms (https://www.oracle.com/java/). The “c2.cp.kegg.v6.2.symbols.gmt gene sets” are selected from the MSigDB (http://software.broadinstitute.org/gsea/downloads.jsp) as the reference gene sets.

### 2.13 Statistical analysis

Groups' differences were determined through Student's *t*‐test or one‐way analysis of variance (ANOVA). The associations between two variables were mainly assessed by Spearson's analysis. All statistical analysis was conducted GraphPad prism 7.0 software (GraphPad software, USA). The *p* < 0.05 was regarded to be statistically significant. * represents *p* < 0.05, ** represents *p* < 0.01, and *** represents *p* < 0.001.

## 3 RESULTS

### 3.1 Bioinformatic analysis shows that SETDB2 expresses lowly in LUAD versus normal tissues and is a prognostic factor

**FIGURE 1:**
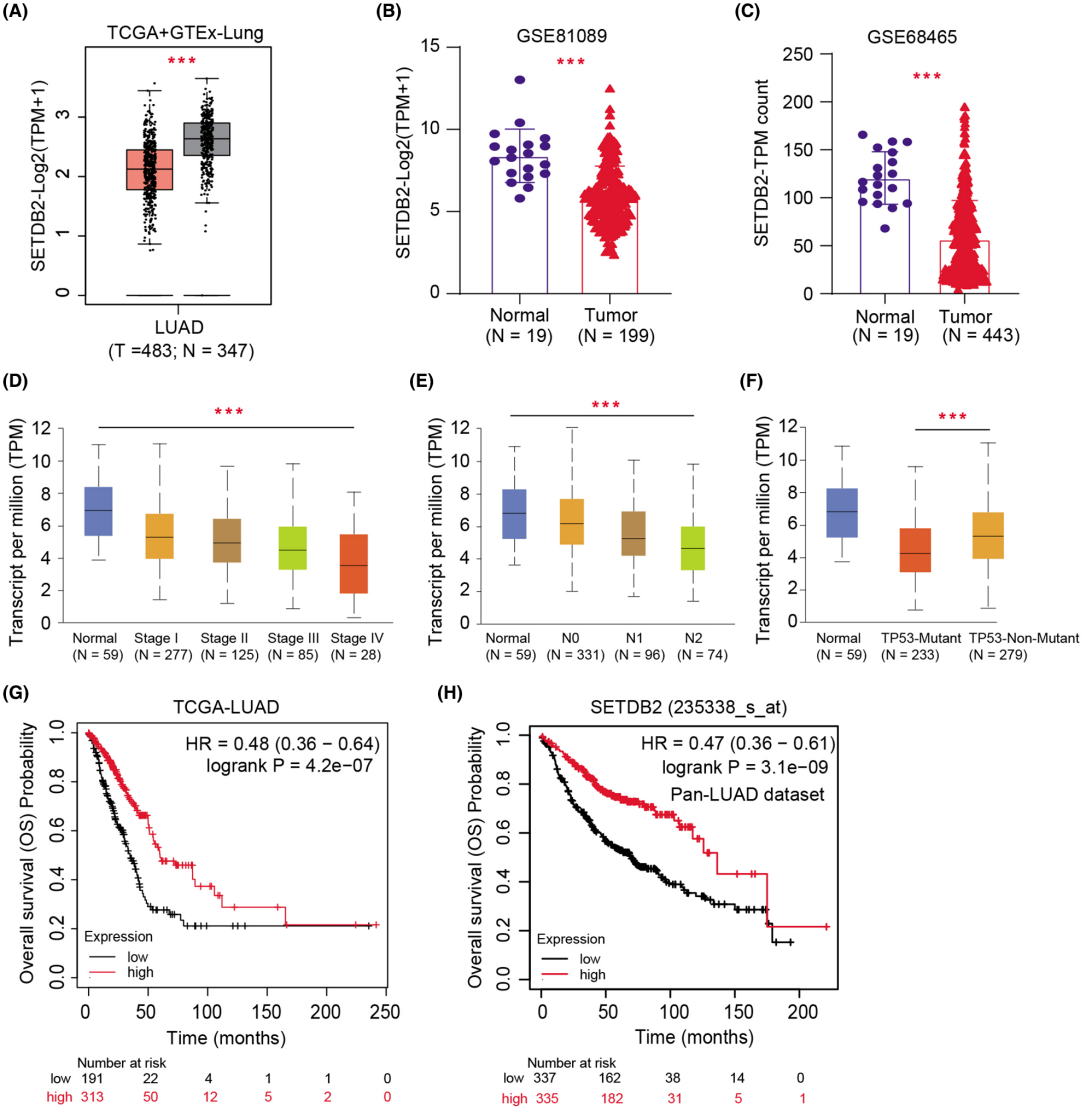
Downregulation of SETDB2 indicates poor prognostic outcomes in LUAD. (A) Differential analysis of SETDB2 mRNA levels in LUAD cohort samples relative to normal samples. (B‐C) Differential analysis of SETDB2 mRNA levels in GSE81089 (*N* = 199) and GSE68465 (*N* = 443). (D‐E) SETDB2 mRNA levels correlated negatively with pathological stages and N stage. (F) Low SETDB2 also correlated with TP53‐mutant phenotype. (G) Kaplan–Meier analysis indicated that patients with low SETDB2 suffered from worse overall survival (OS) outcomes relative to those with high SETDB2 levels (Log‐rank test *p* = 4.2 e‐07).

First, we analyzed the expression data of LUAD samples (*N* = 483) and normal lung tissues (*N* = 347) from the TCGA‐LUAD dataset. The clinical characteristics of LUAD patients from TCGA were summarized in Table S1. We extracted the mRNA levels of SETDB2 and found that SETDB2 was notably downregulated in LUAD samples than normal samples with *p* < 0.001 based on the differential analysis (Figure 1A). Furthermore, we confirmed this result in another two LUAD datasets, including LUAD‐EAS from cBioPortal (https://www.cbioportal.org/) and GSE72094 (https://www.ncbi.nlm.nih.gov/geo/query/acc.cgi) (Figure 1B,C). Meanwhile, we conducted the correlation analysis and found that SETDB2 mRNA levels were negatively associated with hazard clinical characteristics, including pathological stages, nodal metastatic status, and TP53 status (Figure 1D,F). Lastly, we conducted the Kaplan–Meier analysis and found that patients with low SETDB2 mRNA levels had worse prognosis with lower overall survival (OS) rates than those with high SETDB2 mRNA levels (log‐rank test *p* < 0.0001) (Figure 1G).

### 3.2 SETDB2 expression is decreased in collected LUAD samples

**FIGURE 2:**
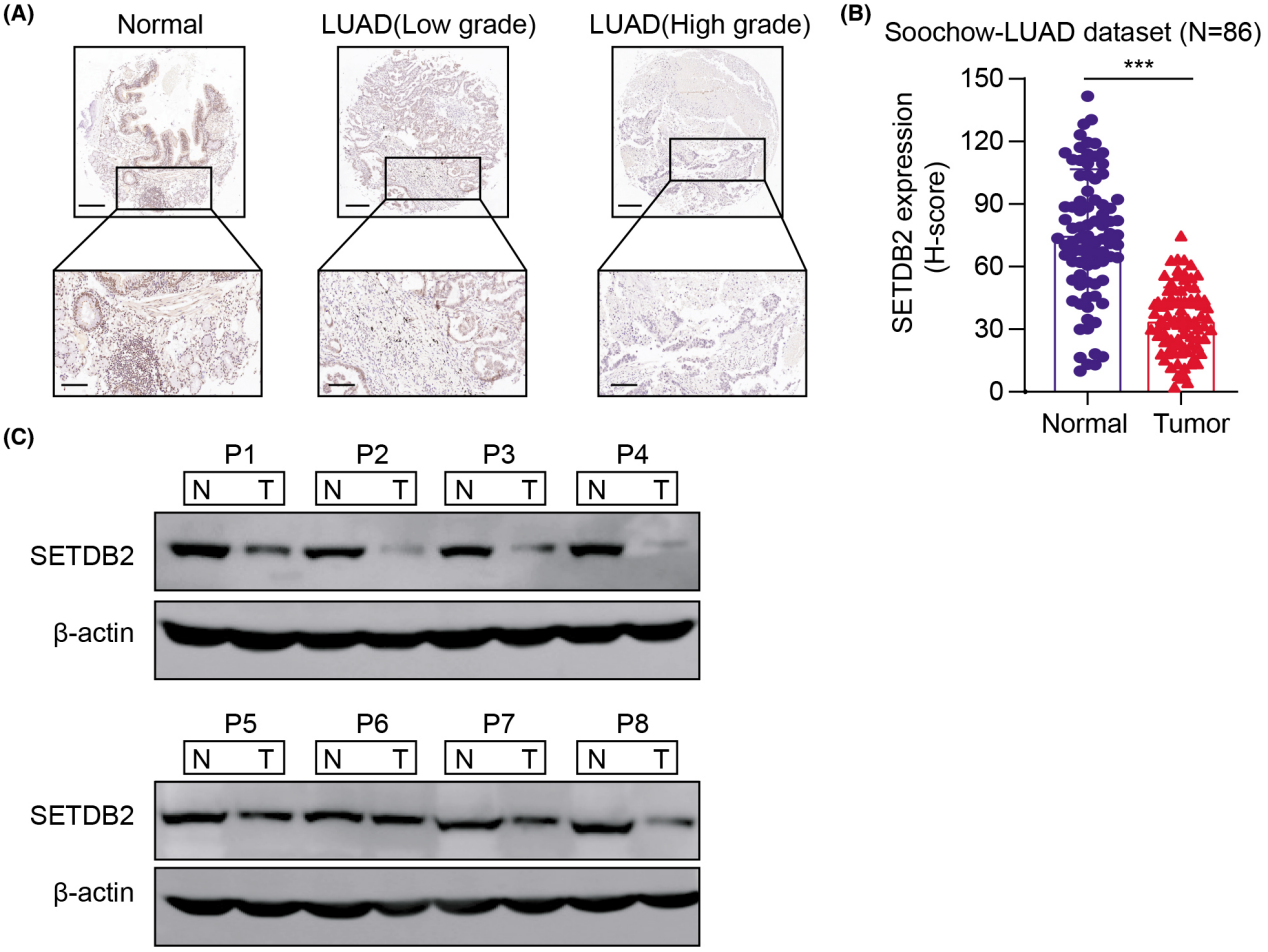
Validation of SETDB2 expression levels in an independent Soochow‐LUAD dataset. (A) Representative IHC staining showed that SETDB2 protein levels were notably downregulated in high‐grade LUAD samples compared with low‐grade tumor samples or normal tissues. (Upper scale bar = 200 μm, lower scale bar = 50 μm). (B) Quantification of IHC scores in tumor samples and normal tissues in Soochow‐LUAD dataset (*N* = 86). (C) SETDB2 protein levels were detected in fresh LUAD tissues and paired normal lung epithelium tissues via western blot (*N* = 8).

Given the findings based on high‐throughput sequencing data from large LUAD samples, we further collected 86 LUAD samples with matched normal tissues to validate the results from our hospital and defined it as the Soochow‐LUAD cohort. We confirmed the speculation in the tissues of patients with LUAD, where SETDB2 protein levels were significantly downregulated in high‐grade LUAD tumor sections compared with low‐grade samples or normal lung tissues via immunohistochemistry (IHC) staining method (Figure 2A,B). We also demonstrated this finding in our fresh LUAD samples via western blot, in which the protein levels of SETDB2 were observed to be remarkably lower in 6/8 (75%) human LUAD tissues than in their matched normal tissues (Figure 2C). Furthermore, tumors in P1–4 belong to patients with high grades, and P5–8 belong to patients with low grades (Figure 2C). Taken together, the above results revealed that SETDB2 mRNA levels were significantly downregulated in LUAD samples, which was a prognostic factor.

### 3.3 SETDB2 inhibition promotes tumor growth, migration, and stemness features of LUAD

**FIGURE 3:**
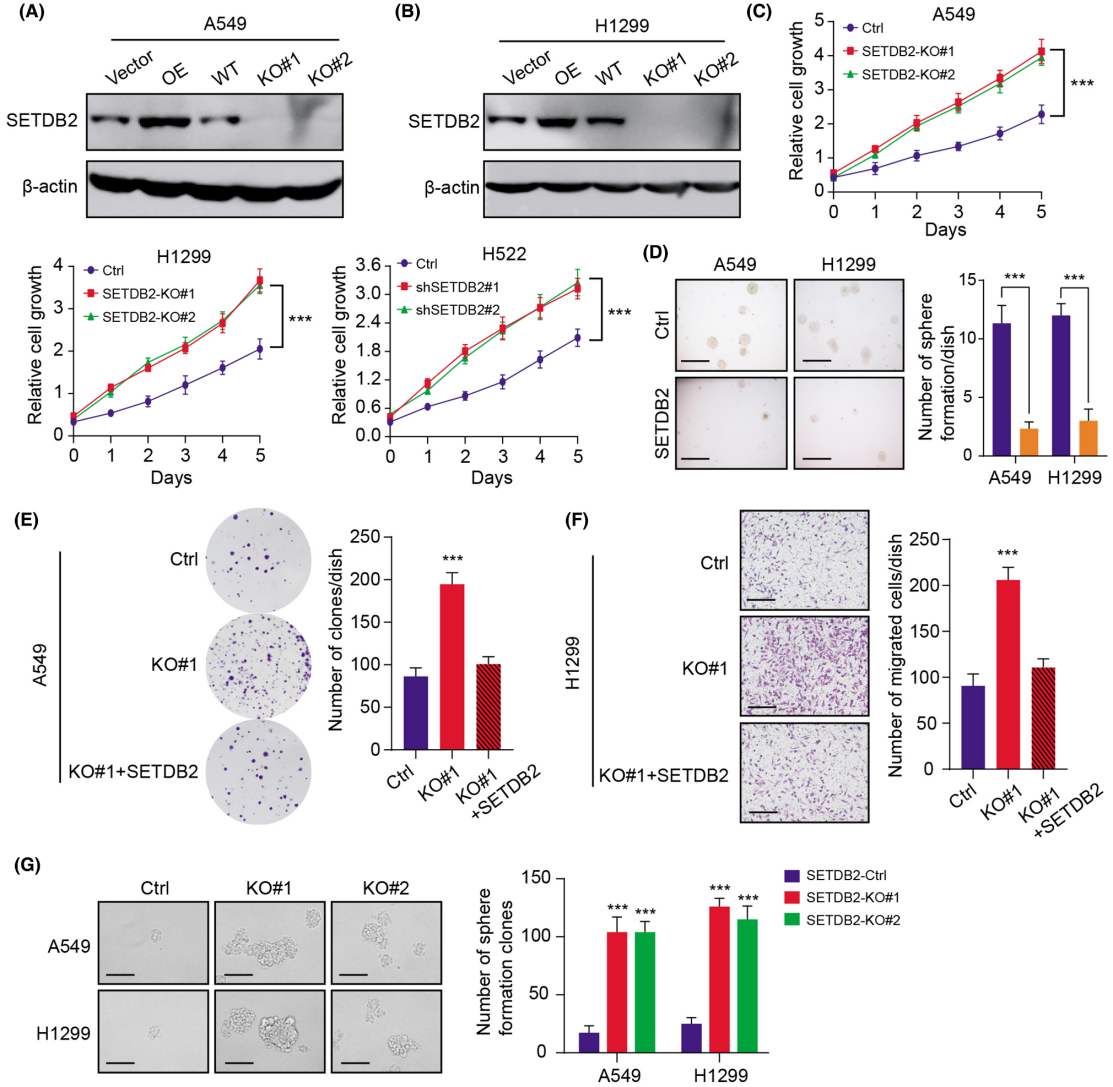
Low SETDB2 enhances tumor growth, migration, and stemness features of LUAD cells. (A‐B) The protein levels of SETDB2 in A549 and H1299 with SETDB2 overexpression or knockout were determined by western blot. (C) Relative to those parental control cells, SETDB2 knockout or knockdown could significantly promote cell growth rate determined by CCK‐8 method. (D) SETDB2 overexpression could significantly enhance LUAD cell (A549 and H1299) anchorage‐independent growth in soft agar (scale bars = 250 μm, left panel). Quantification of colony formation experiments were exhibited on the right panel. (E) The clonogenic ability in A549 cells was elevated with SETDB2 deficiency, which could be repressed with wild‐type SETDB2. (F) Similarly, SETDB2 knockout enhanced migration ability of H1299, which could be repressed with SETDB2 overexpression. Scale bar = 200 μm. (G) Representative pictures and quantification of the sphere‐formation assay of SETDB2 knockout LUAD cells and control cells (*N* = 6). Scale bar = 200 μm

Considering that we have already found the significantly downregulated levels of SETDB2 in LUAD, the functional role of SETDB2 still remained unclear. We queried the expression levels of SETDB2 in the Cancer Cell Line Encyclopedia (CCLE) dataset and selected three human lung adenocarcinoma cell lines (A549, H1299, and H522) to conduct the experimental assays (Table S2). We utilized the *CRISPR/Cas9* technology using specific sgRNAs to knockout SETDB2 in A549 and H1299, which was confirmed via western blot (Figure 3A,B). Meanwhile, we overexpressed the SETDB2 tagged with GFP via lentivirus infection method (Figure 3A,B). Then, we explored that whether SETDB2 could influence the proliferation of LUAD cells. We found that the growth capacity of cell lines (A498, H1299, and H522) was dramatically increased following SETDB2 deficiency or knockdown based on the CCK8 assays, relative to those parental control cells (Figure 3C). Additionally, SETDB2 overexpression remarkably suppressed the growth of LUAD cells through the soft agar colony formation assay (Figure 3D). In addition, SETDB2 deficiency could remarkably increase the growth efficiency of A549 cells, which could be completely reversed with the restoration of SETDB2 (Figure 3E). Similarly, SETDB2 knockout also enhanced the migration ability of A549, which could be suppressed upon SETDB2 overexpression (Figure 3F). The colony formation assay and transwell assay were further repeated in another H522 cell line, and we observed the similar results (Figure S1A). Lastly, SETDB2 knockout cells exhibited increased capacity of sphere formation, indicating the suppressive role of SETDB2 in regulating LUAD self‐renewal and stemness capacities (Figure 3G). Collectively, our findings indicated that SETDB2 functions as a tumor suppressor in LUAD and SETDB2 inhibition could promote the capacities of cell proliferation, migration, and stemness.

### 3.4 SETDB2 correlates with oxidative stress and epigenetically suppresses downstream NRF2 transcription

**FIGURE 4:**
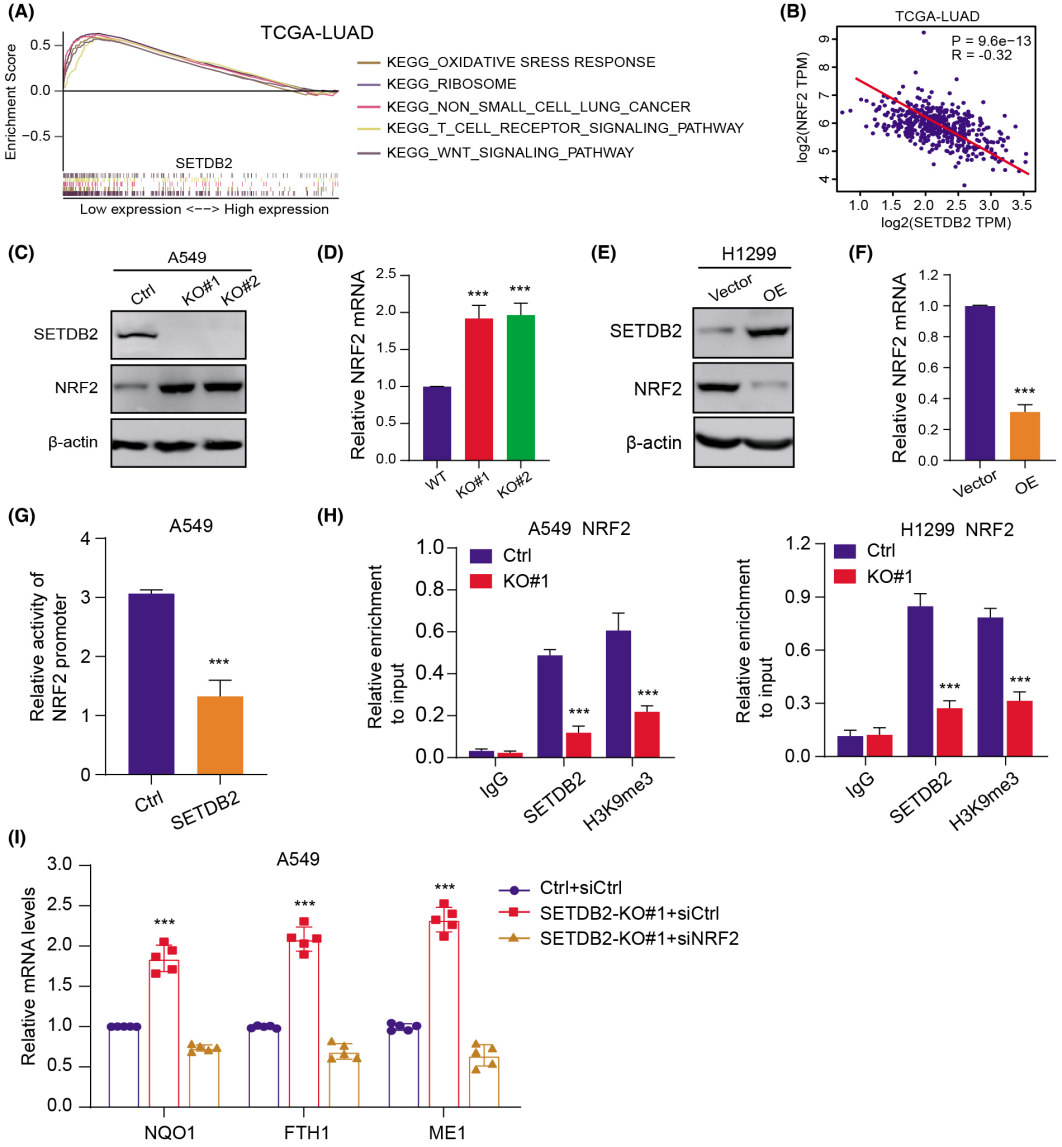
SETDB2 epigenetically suppresses NRF2 expression. (A) Gene Set Enrichment Analysis (GSEA) indicated that samples with low SETDB2 were mainly enriched with several oncogenic crosstalk, including oxidative stress responses, WNT signaling pathway, and T cell receptor signaling pathway. (B) Correlation analysis suggested that SETDB2 correlated negatively with NRF2 mRNA levels in TCGA‐LUAD dataset (*Pearson r* ^2^ = −0.32, *p* = 9.6 e‐13). (C) NRF2 expression levels were notably elevated upon SETDB2 knockout, which was detected by western blot (*N* = 3). (D) Elevated SETDB2 mRNA levels were found in SETDB2‐deficient A549 relative to control cells (*N* = 3). (E‐F) Accordingly, SETDB2 overexpression could significantly suppress the protein levels of SETDB2 (*N* = 3). (E) and corresponding mRNA levels (F). (G) Assessment of NRF2 promoter activity after SETDB2 overexpression in A549 cells via dual‐luciferase reporter assay (*N* = 3). (H) ChIP‐PCR was conducted to confirm the co‐occupancy of SETDB2 and H3K9me3 modification at the promoter of NRF2 in A549 and H1299 cells with deficient SETDB2 and wild‐type SETDB2, in which IgG was a negative control (*N* = 3). (I) SETDB2 deficiency could significantly elevate the mRNA levels of representative NRF2‐downstream targets (NQO1, FTH1, and ME1), which could be repressed with NRF2 knockdown (*N* = 5).

First, the Gene set enrichment analysis (GSEA) exhibited that SETDB2 and oxidative stress crosstalk were highly interrelated based on the expression data from TCGA‐LUAD cohort (Figure 4A). It is well documented that aberrant NRF2 activation is a critical driver and represents a molecular hallmark in LUAD, functioning as a master regulator of anti‐oxidative stresses. Intriguingly, we observed the significantly negative associations between NRF2 and SETDB2 mRNA levels in the TCGA‐LUAD samples (Figure 4B). We detected that SETDB2 deficiency could elevate NRF2 mRNA and protein levels (Figure 4C,D), whereas SETDB2 overexpression significantly suppressed the mRNA and protein levels of NRF2 (Figure 4E,F). We thus hypothesized that SETDB2 may influence the transcriptional activity of NRF2, which altered its mRNA and subsequent protein levels. To confirm our speculations, we conducted the dual luciferase reporter assay in A549 cells, which showed that SETDB2 overexpression may suppress the activity of NRF2 promoter (Figure 4G). Next, the chromatin immunoprecipitation (ChIP) assay was conducted using SETDB2 and H3K9me3 antibodies and we further designed specific primers of NRF2 promoter to assess the NRF2 enrichment. We observed that the NRF2 promoter sequences simultaneously enriched by the two antibodies were remarkably decreased upon SETDB2 ablation relative to control group (Figure 4H). Lastly, we performed the RT‐qPCR method to find that the mRNA levels of representative NRF2 downstream targets (NQO1, FTH1, and ME1) were profoundly increased in SETDB2‐knockout cells, which could be reduced upon NRF2 knockdown. These results implicated that SETDB2 could bind to NRF2 promoter and promote the enrichment of H3K9me3 to inhibit the mRNA levels of NRF2. Collectively, this study found that SETDB2 regulates oxidative crosstalk and could epigenetically restrain NRF2 expression levels.

### 3.5 Downregulated SETDB2 depends on NRF2 to drive malignant processes of LUAD

**FIGURE 5:**
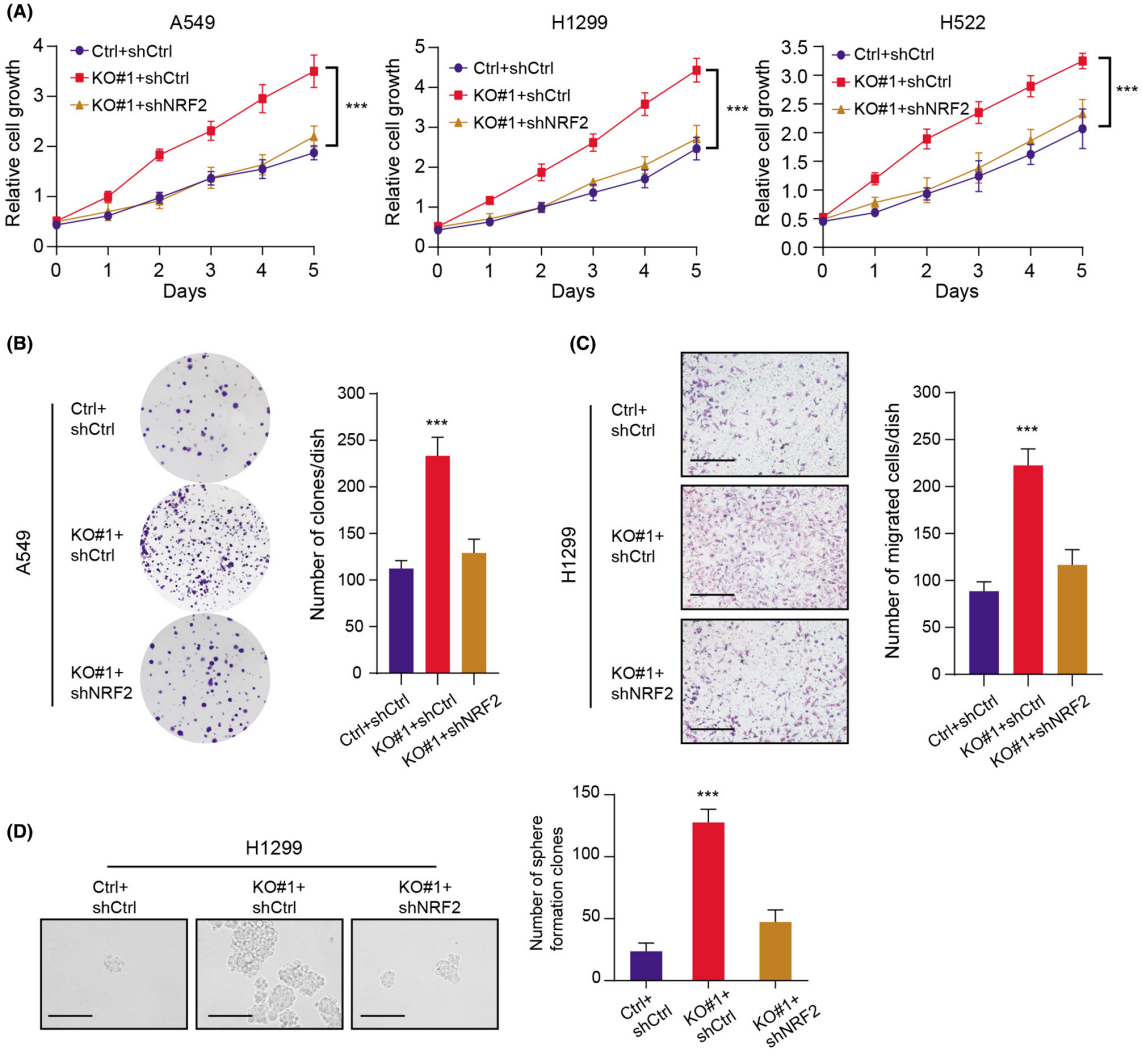
Low SETDB2 depends on NRF2 to drive malignant process in LUAD cells. (A) NRF2 knockdown significantly inhibited the cell growth capacity caused by SETDB2 deficiency in three independent cell lines (A549, H1299, and H522) (*N* = 3). (B) Representative images (scale bars = 200 μm, left panel) and quantification results (right panel) of the cell cologenic abilities of SETDB2‐deficient A549 cells transfected with NRF2 shRNAs or their corresponding controls (*N* = 3). (C) SETDB2 knockout could remarkably elevate the migration ability of H1299 cells, which could be repressed by NRF2 inhibition (*N* = 3). Scale bar = 200 μm. (D) Similarly, SETDB2 deficiency could significantly enhance the sphere formation of H1299 cells, which could be repressed by NRF2 knockdown (*N* = 3). Scale bar = 200 μm.

To further characterize the functional associations between SETDB2 and NRF2 in LUAD, we designed two different siRNAs to target NRF2 in SETDB2‐deficient cells. As expected, knockdown of NRF2 markedly suppressed the growth capacity induced by SETDB2 ablation, which was confirmed by three independent cell lines (Figure 5A). Similarly, the enhanced colony formation efficiency, migration ability, and self‐renewal process in SETDB2‐knockout cells were all profoundly suppressed with NRF2 inhibition (Figure 5B‐D). Lastly, the NRF2‐driving tumor‐promoting abilities could be largely suppressed via ectopic overexpression of SETDB2, as indicated by the CCK‐8 assays (Figure S1B). As a result, our data suggested that SETDB2 deficiency promotes LUAD malignant processes at least partially depending on accumulated NRF2 levels.

### 3.6 SETDB2/NRF2 axis correlates with chemotherapy sensitivity in LUAD and has clinical significance

**FIGURE 6:**
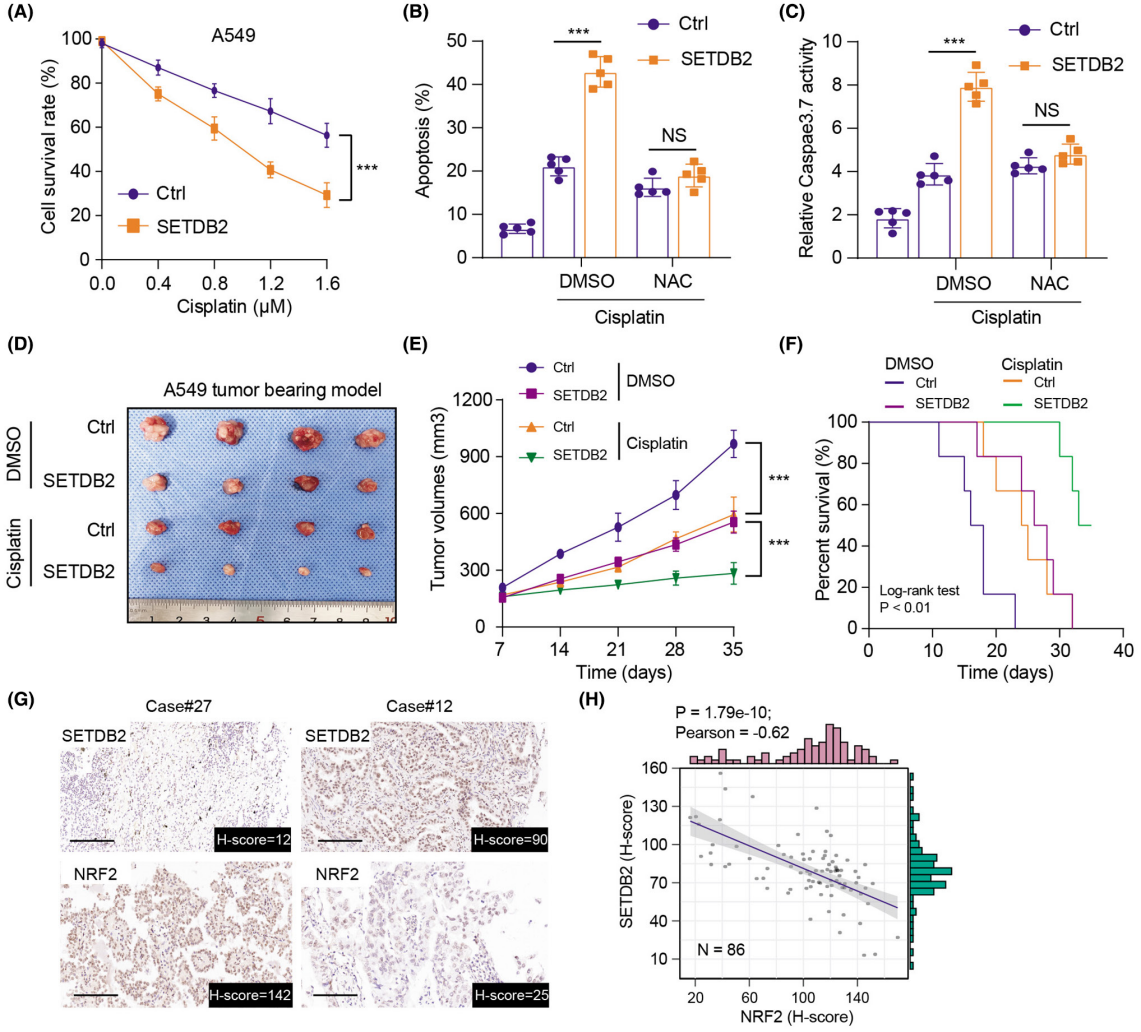
SETDB2/NRF2 crosstalk was associated with chemotherapy sensitivity in LUAD cells and has clinical significance. (A) The sensitivity of A549 cells to Cisplatin treatment was remarkably elevated upon SETDB2 overexpression according to the half maximal inhibitory concentration (IC50) values (*N* = 3). (B‐C) In line with the effect found under basal conditions, a more evident proapoptotic phenomenon of SETDB2‐OE was observed in the presence of Cisplatin treatment (1.2 μM), suggesting that SETDB2 overexpression allows cells to resensitize to Cisplatin‐induced apoptosis (*N* = 5). (D‐F) Consistent with in vitro findings, the subcutaneous tumor model further revealed that SETDB2‐OE had synergistic effect with Cisplatin treatment, as quantified by tumor volumes (D‐E) and Kaplan–Meier analysis (F). (G) Representative pictures showed the IHC staining of SETDB2 and NRF2 expression levels. (H) Correlation analysis confirmed the negative associations between SETDB2 and NRF2 in tumor samples from Soochow‐LUAD dataset.

It has been well known that cellular ROS is essential for the chemotherapy response among various tumors, especially in LUAD. Based on the robust associations between SETDB2‐ and NRF2‐mediated anti‐oxidative process, we further examined that whether SETDB2 overexpression could improve the chemotherapy sensitivity in LUAD. Indeed, we first found that cisplatin inhibited cell growth more dramatically than in SETDB2‐overexpression cells than controls (Figure 6A). As is well documented, N‐acetyl‐cysteine (NAC), one cellular reductant, could be effective to antagonize ROS within cells and indirectly reflect its levels. Thus, both DNA damage and apoptosis induced by SETDB2‐OE could be remarkably obliterated by NAC in cells treated with cisplatin, indicating the significance of accumulated ROS levels for SETDB2‐OE‐mediated apoptosis in LUAD cells (Figure 6B,C). In line with our in vitro results, the subcutaneous tumor models further revealed that cisplatin had a mild inhibitory effect on the growth of tumors derived from SETDB2‐WT A549 cells. However, SETDB2‐OE significantly suppressed the tumor volumes and exhibited profound synergistic effect with cisplatin treatment, as indicated by tumor volumes (Figure 6D,E). Kaplan–Meier analysis further suggested that mice from the fourth group (SETDB2‐OE + cisplatin) had an improved prognosis than the other three groups with *p* < 0.0001, suggesting that SETDB2 overexpression has the synergistic effect with cisplatin for LUAD tumors (Figure 6F). Lastly, the robust associations of SETDB2 and NRF2 levels were further confirmed in clinical LUAD samples via IHC, and we found that SETDB2 levels correlated negatively with NRF2 levels (Figure 6G‐H). These IHC graphs showed these patients representative of SETDB2^high^/NRF2^low^ and SETDB2^low^/NRF2^high^. Taken together, these findings suggested that SETDB2 overexpression could be able to improve the chemotherapeutic efficacy of cisplatin via elevating ROS levels in LUAD through inhibition of NRF2.

## 4 DISCUSSION

In recent years, great progression has been made in the treatment of LUAD, however, the overall prognosis of patients still remains unfavorable.^35^ Epigenetic drivers play essential roles in tumor biology, and accumulating evidence suggests that multiple epigenetic factors participate in manipulating the tumor progression or distal metastasis.^36^, ^37^ The transcription factor PR domain containing 16 (PRDM16) was found to be downregulated in lung adenocarcinomas, which represses the transcription of Mucin‐4 (MUC4), one of the regulators of epithelial‐to‐mesenchymal transition (EMT) process of cancer cells.^38^ The histone reader protein HP1γ could modulate the epigenetic suppression of the transcription‐repressive regulators NCOR2 and ZBTB7A to activate the protumorigenic transcriptome in lung adenocarcinoma.^39^ In addition, histone deacetylase SIRT1 regulates the mitochondrial oxidative phosphorylation (mtOXPHOS) system to impact the sensitivity of tyrosine kinase inhibitor (TKI) in LUAD.^40^ In this study, we identified a novel role of epigenetic repressor, SETDB2, in the tumorigenesis of LUAD. Previous studies have identified that SETDB2 functions as a transcriptional repressive factor in leukemia, which is consistent with its contributions in multiple homeostatic deseases.^24^ SETDB1 and SETDB2 belong to the members of the SUV39 family of lysine methyltransferases, also including SETD8, GLP, SUV39H1, or G9a. Distinct from other members, SETDB2 and SETDB1 both contain a methyl‐CpG‐binding domain, which promotes the recruitment of H3K9me3 at nearby histones. As is well known, histone methylation at H3K9 by SET domain‐containing proteins play essential roles to regulate developmental process and genomic stability. Previous studies have revealed that H3K9me3 shows different dynamic characteristics in promoters and long terminal repeats (LTRs), promoting dramatic reprogramming during early stage of embryonic development.^41^ Moreover, researchers have demonstrated that H3K9me3‐mediated epigenetic regulation of senescence could predict survival outcome of lymphoma patients.^42^

In this study, we first demonstrated that SETDB2 could link the associations between H3K9me3 with oxidative stress in tumor progression. Different from oncogenic roles in breast cancer, gastric cancer, or leukemia, we identified that SETDB2 is a tumor repressor in LUAD, which expressed lowly in tumor samples. Functional assays confirmed that SETDB2 deficiency could accelerate the malignant processes, including cell proliferation, migration, or stemness maintenance. SETDB2 levels correlated negatively with NRF2 and its downstream targets. ChIP assays confirmed that SETDB2 mainly mediates the enrichment of H3K9me3 at the promoter region of NRF2, repressing its transcriptional expressions. As a result, downregulated SETDB2 in LUAD contributed to the accumulation and activation of NRF2, thereby enhancing the downstream anti‐oxidative crosstalk. Besides, low levels of SETDB2 depended on NRF2 to drive the malignant features of LUAD. Given the tight associations between SETDB2 and NRF2 crosstalk, we further found that SETDB2 overexpression could further attenuate the NRF2 pathway and enhance the sensitivity of chemotherapy via upregulating cellular ROS levels. The SETDB2 expression could be used as the indicator of chemotherapy sensitivity for LUAD patients and the SETDB2/NRF2 axis could be biomarkers for predicting prognosis.

Activation of oxidative stress response represents a biological hallmark in tumorigenesis and aggressiveness of LUAD, which is also commonly observed in other malignant tumors.^43^ NLUCAT1 is a large nuclear transcript and repressed genes within the antioxidant or cisplatin‐response networks, which represented a novel therapeutic target in LUAD.^44^ Adenylate kinase 4 (AK4) was reported to modulate oxidative stress that enhanced lung cancer metastasis.^45^ Besides, the aldehyde dehydrogenase 1A1 (ALDH1A1) confers erlotinib resistance by facilitating the ROS‐RCS metabolic crosstalk, highlighting the robust associations between oxidative stress and drug resistance.^46^ It was well known that KEAP1 mutated frequently in LUAD that contributes to the accumulation of NRF2 protein levels, which is the essential mechanism identified to explain the activation of NRF2 signaling. For instance, DPP3 is overexpressed and binds KEAP1 in breast cancer, and enhanced—DPP3‐KEAP1 interaction could significantly attenuate the ubiquitination‐mediated degradation of NRF2.^47^ Meanwhile, inhibitor of Apoptosis Stimulating Protein of p53 (iASPP), could compete with NRF2 for KEAP1 binding with a DLT motif, which resulted in NRF2 accumulation and anti‐oxidative transactivation.^33^ Different from the mechanisms of posttranslational modification like ubiquitination, we identified that SETDB2 could directly regulate the NRF2 expression levels via recruiting H3K9me3 enrichment at the promoter region. Investigations of epigenetic control of NRF2 remained relatively less in tumor, but it has pivotal clinical significance. Researches have already found that 5‐Aza/TSA treatment could significantly restore the expression levels of NRF2 via decreasing the levels of DNMT, H3K9me3, and MeCP2 at the NRF2 promoter.^34^ How to target SETDB2 to elevate its expression levels would receive multiple efficacy, including suppression of tumor growth and improvement of chemotherapy sensitivity in LUAD.

However, despite the intriguing findings, several limitations also existed that need to be dealt with in the following studies. First of all, large tumor samples were warranted to conduct IHC assay to further evaluate the prognostic significance of SETDB2 in LUAD. Second, apart from downstream NRF2 signaling crosstalk, whether there existed other biological items regulated by SETDB2, like immune invasion, or autophagy, that contribute to LUAD tumor progression remains unclear. Large high‐throughput sequencing methods, including ChIP‐seq and RNA‐seq, are effective to systematically screen SETDB2 downstream targets. In addition, the GSEA revealed that samples with low SETDB2 expressions were also enriched with other signaling, like the WNT signaling pathway. Activation of WNT signaling contributes to Epithelial‐to‐Mesenchymal (EMT) transition and stemness in lung cancer. However, owing to limitations, it remains unclear that how SETDB2 regulates WNT signaling and we would clarify this point in the following studies. Lastly, given the clinical significance of SETDB2, how to define the rational cutoff that discriminates SETDB2^high^ and SETDB2^low^ samples would be essential for guiding individual chemotherapy in LUAD patients.

## 5 CONCLUSION

In summary, this study identified that SETDB2 expressed lowly and functioned as a tumor suppressor in LUAD. SETDB2 recruited the H3K9me3 at the NRF2 promoter to restrain its transcriptional levels. Downregulated SETDB2 promoted and depended on accumulated NRF2 to manipulate downstream oxidative crosstalk. SETDB2 overexpression could render LUAD sensitive to cisplatin treatment, providing a therapeutic target.

## AUTHOR CONTRIBUTIONS

**Guangda Yuan:** Data curation (lead); formal analysis (lead); methodology (equal). **Bowen Hu:** Investigation (equal); methodology (equal). **Jun Ma:** Data curation (equal); formal analysis (equal); funding acquisition (equal). **Chuanyu Zhang:** Formal analysis (equal); resources (equal). **Hongya Xie:** Data curation (equal); formal analysis (equal); investigation (equal). **Tengteng Wei:** Formal analysis (equal); methodology (equal); resources (equal); software (equal); supervision (equal). **Yong Yang:** Conceptualization (equal); writing – original draft (equal). **Bin Ni:** Conceptualization (equal); writing – review and editing (equal).

## CONFLICT OF INTEREST

The authors declare that the research was conducted in the absence of any commercial or financial relationships that could be construed as a potential conflict of interest.

## ETHICS STATEMENT

The human tissue specimens and clinical data were reviewed and approved by the First Affiliated Hospital of Soochow University (Jiangsu, China) with the approval number of IEC‐C‐006‐A17. All patients have signed the written informed consent permitted by the Ethics Review Committee of the First Affiliated Hospital of Soochow University (Jiangsu, China).

## Supporting information


Figure S1.

Table S1.

Table S2.

## Data Availability

The molecular experiment data generated and analyzed during the current study are available from the corresponding author on reasonable request.
