# Supplementary figures and images for: Histone lysine methyltransferase SETDB2 suppresses NRF2 to restrict tumor progression and modulates chemotherapy sensitivity in lung adenocarcinoma
Source: Cancer Med. 2022 Dec 12;12(6):7258–72. doi: 10.1002/cam4.5451 (PMC10067124; doi:10.1002/cam4.5451)

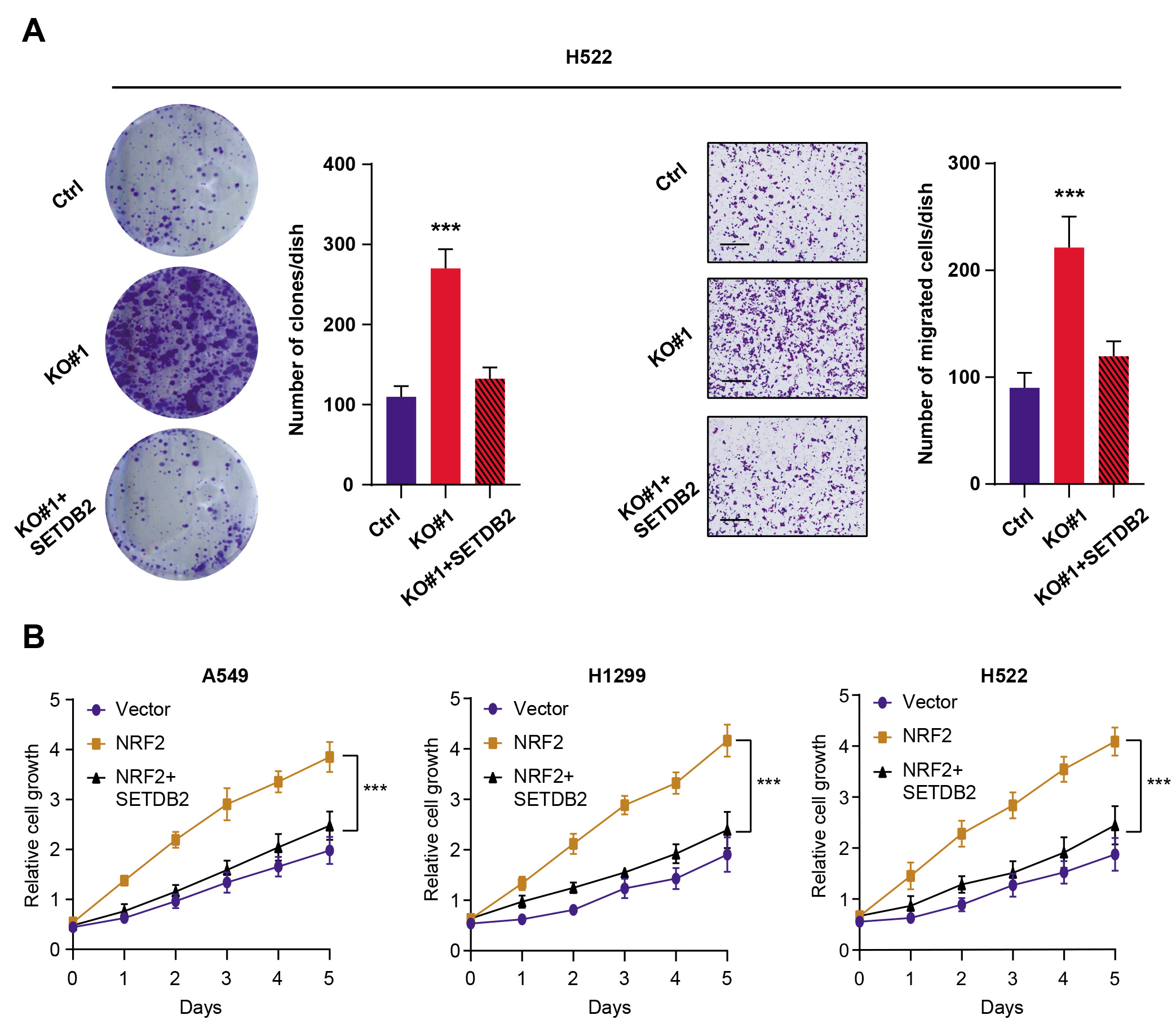

Supplement: Supplementary file 1 — Figure S1. [file CAM4-12-7258-s002.jpg]
